# Flexibility of a Eukaryotic Lipidome – Insights from Yeast Lipidomics

**DOI:** 10.1371/journal.pone.0035063

**Published:** 2012-04-18

**Authors:** Christian Klose, Michal A. Surma, Mathias J. Gerl, Felix Meyenhofer, Andrej Shevchenko, Kai Simons

**Affiliations:** Max Planck Institute of Molecular Cell Biology and Genetics, Dresden, Germany; Texas A&M University, United States of America

## Abstract

Mass spectrometry-based shotgun lipidomics has enabled the quantitative and comprehensive assessment of cellular lipid compositions. The yeast *Saccharomyces cerevisiae* has proven to be a particularly valuable experimental system for studying lipid-related cellular processes. Here, by applying our shotgun lipidomics platform, we investigated the influence of a variety of commonly used growth conditions on the yeast lipidome, including glycerophospholipids, triglycerides, ergosterol as well as complex sphingolipids. This extensive dataset allowed for a quantitative description of the intrinsic flexibility of a eukaryotic lipidome, thereby providing new insights into the adjustments of lipid biosynthetic pathways. In addition, we established a baseline for future lipidomic experiments in yeast. Finally, flexibility of lipidomic features is proposed as a new parameter for the description of the physiological state of an organism.

## Introduction

One of the central problems in cellular lipid physiology is to understand how hundreds of existing lipids representing an enormous structural diversity contribute to cell function. Over the past several years lipidomic studies of various experimental systems and model organisms have begun to generate insights into the role of lipids in various processes on the cellular and organismal level [1]–[7]. The yeast *Saccharomyces cerevisiae* has proven to be a particularly valuable and widely used experimental system for studying lipid-related processes. Studies with yeast have provided new insights into neutral lipid metabolism, sphingolipid homeostasis, physiological sphingolipid-sterol interactions as well as the integration of lipid metabolism with cell cycle signals and cellular proliferation [4], [8]–[11]. The recent development of mass spectrometry-based shotgun lipidomics has made it possible to achieve a comprehensive and quantitative assessment of cellular lipidomes covering neutral lipids, glycerophospho- (GP) as well as sphingolipids (SP) and sterols in the course of a single experiment [1], [4].

The lipid composition of yeast cells is relatively simple compared with more complex multicellular organisms, yet its major features are comparable to the lipidome of any other eukaryotic cell. The most abundant membrane lipid classes are: phosphatidic acid (PA), phosphatidylcholine (PC), phosphatidyl-ethanolamine (PE), phosphatidylinositol (PI), phosphatidylserine (PS), diacylglycerol (DAG) and their respective lyso-derivatives. Less abundant are phosphatidylglycerol (PG) and cardiolipin (CL). Triacylglycerols (TAG) and sterol esters (SE) serve as storage lipids. The fatty acid (FA) composition of these classes is mostly limited to palmitic (C16:0), palmitoleic (C16:1) and oleic acid (C18:1), with only minute amounts of shorter FAs present. The major yeast sterol is ergosterol (Erg). The yeast sphingolipids (SP) consist of inositolphosphorylceramide (IPC), mannosyl-inositol phosphorylceramide (MIPC) and mannosyl-di-(inositolphosphoryl) ceramide (M(IP)_2_C). Their ceramide backbone contains phytosphingosine as the long chain base (LCB) moiety to which a very long chain fatty acid (VLCFA), mostly α-hydroxylated C26:0, is attached via an amide linkage.

The yeast lipidome has been qualitatively and quantitatively described in great detail [1], [12], [13]. Prior to this work, studies had focused primarily on the overall FA composition but not the FA composition of particular lipid species (e.g. [14]). Additionally, much of the research has focused on GP and sterols at the expense of other lipid classes, especially SP. In examining the published data and our own results, we noticed a substantial degree of variability in the amounts of the different lipids in yeast. We hypothesized that this lipidomic variability reflects intrinsic flexibility, i.e. the natural ability of a lipidome to undergo changes in response to different growth conditions. The extents to which a eukaryotic lipidome can be reshaped as well as the contribution of different lipids to this flexibility have never been investigated. In order to gain insight into this lipidomic flexibility, we monitored how the yeast lipid composition changes as a function of growth conditions.

To this end, we systematically investigated the influence of various growth conditions, like temperature, carbon source, over-expression of different proteins and growth phase on the lipidome of yeast cells. We generated a detailed catalogue of yeast lipidomes under the various conditions, establishing a baseline for future experiments in yeast. In order to handle and interpret the large volume of collected data, we developed a novel, lipidomic feature-based approach, which enabled a rapid processing of this extensive dataset, thus facilitating data interpretation. Importantly, this approach enabled a quantitative assessment of the flexibility of the various lipidomic features and provides insights into the natural variability of a eukaryotic cellular lipidome.

## Materials and Methods

### Yeast strains and plasmids

Yeasts used were the standard laboratory wild-type *Saccharomyces cerevisiae* strains: BY4741 *MATa his3*Δ1 *leu2*Δ0 *met15*Δ0 *ura3*Δ0; BY4742 *MATα his3*Δ1 *leu2*Δ0 *lys2*Δ0 *ura3*Δ0; BY4743 *MATa/α his3*Δ1/*his3*Δ1 *leu2*Δ0/*leu2*Δ0 *lys2*Δ0/*LYS2 MET15*/*met15*Δ0 *ura3*Δ0/*ura3*Δ0 (4741/4742); and knock-out *dan2*Δ strain *MATa his3*Δ1 *leu2*Δ0 *met15*Δ0 *ura3*Δ0 *dan2*Δ::KAN^r^, all purchased from Open Biosystems.

All plasmids were based on the p41XGALS backbone, which is a yeast expression, galactose-inducible promoter-containing centromere plasmid [15]. pGFP contains eGFP in p416GALS; pTM contains FusMidGFP in p416GALS [16].

### Media

All media were prepared as described [17], using Difco yeast nitrogen base w/o amino acids (BD), Bacto peptone (BD), Bacto yeast extract (BD), complete (or drop-out) supplement mixture (MP Biomedicals) and the indicated carbon source (all molecular biology purity, Merck) , and G418 (Formedium).

Complete media used: YPglc (yeast extract/peptone/glucose 2%), YPraf (raffinose 2%), YPgly (glycerol 3%). Synthetic complete (SC) media used: SCglc (glucose 2%), SCraf (raffinose 2%). For the expression experiments SCraf was used and the induction was conducted in SCrafgal (raffinose 2%, galactose 2%) medium.

### General experimental procedure

The general experimental procedure for obtaining total cell lipids consisted of the following steps. Yeast from frozen −80°C glycerol stocks were plated on SCglc 2% Bacto agar media (for plasmid transformed and *dan2*Δ strains proper selection plates were used) and grown for 48 h at 30°C. Plated yeasts were used to inoculate 5 ml liquid precultures (triplicated) in media type/temperature used for the tested condition and grown in glass tubes tilted and shaken at 180 rpm for 16 h. Experimental cultures (triplicated) were set using precultured yeast, diluted to OD_600 nm_ 0.2 in 20 ml (inoculation volume did not exceed 5% of culture total volume) of the respective media and grown in 100 ml Erlenmeyer microbiological flasks at 180 rpm and indicated temperature until the desired OD_600 nm_ was reached. Detailed conditions for each experiment are described in further sections. Then yeast cells were pelleted by 3 min centrifugation at 5000 g in 50 ml plastic centrifuge tubes with screw caps, resuspended in 20 ml 155 mM ammonium bicarbonate and spun down similarly. Cell pellets were resuspended in fresh 1 ml ammonium bicarbonate in 2 ml plastic microcentrifuge tubes and spun for 1.5 min at 5000 g. All centrifugation and washing steps were performed at 20°C. After the last wash, supernatant was discarded and pellets were snap-frozen in liquid nitrogen. The entire sample harvesting procedure took in total 10–12 min. Prior to cell lysis, frozen pellets were stored up to one week at −80°C. For cell lysis, pellets were thawed and resuspended in 1 ml ammonium bicarbonate and shaken with 200 µl of zirconia beads in 2 ml eppendorf tubes for 20 min using TissueLyser II (Qiagen) at 4°C. A volume equivalent to 0.2 OD_600 nm_ units was taken and diluted with ammonium bicarbonate to 200 µl.

### Lipid extraction and mass spectrometric analysis

The lipid compositions of total cell extracts were determined by quantitative shotgun lipidomic analysis as previously described [1]. Samples were mixed with 30 µl of internal lipid standard mixture, providing a total spike of 24 pmol DAG 17:0-17:0, 22 pmol PA 17:0-14:1, 41 pmol PE 17:0-14:1, 41 pmol PS 17:0-14:1, 42 pmol PC 17:0-14:1, 40 pmol PI 17:0-14:1, 14 pmol CL 15:0-15:0-15:0-16:1, 22 pmol ceramide 18:0;3/18:0;0, 37 pmol IPC 18:0;2/26:0;0, 36 pmol MIPC 18:0;2/26:0;0, 31 pmol M(IP)_2_C 18:0;2/26:0;0, and 57 pmol cholesterol-D7. Samples were subsequently subjected to two-step lipid extraction (1 ml of solvent in each step) executed at 4°C. The lower organic phases were collected and evaporated in a vacuum evaporator at 4°C. Lipid extracts were dissolved in 100 µl chloroform/methanol (1∶2; vol/vol) and analyzed by mass spectrometry using a LTQ Orbitrap XL (Thermo Fisher Scientific) equipped with a robotic nanoflow ion source TriVersa NanoMate (Advion Biosciences). PA, PS, PE, PC, CL, PI, IPC, MIPC and M(IP)_2_C, DAG and lysolipid species were monitored by negative ion mode FT MS analysis, whereas TAG and ceramide species were monitored by positive ion mode FT MS analysis. Sterols were subjected to chemical sulfation and analyzed by negative ion mode FT MS analysis [18].

### Nomenclature

The following lipid names and abbreviations were used. SP – sphingolipids, include: Cer – ceramides, IPC – inositolphosphorylceramide, MIPC – mannosyl-inositol phosphorylceramide, M(IP)_2_C – mannosyl-di-(inositolphosphoryl) ceramide. GP – glycerophospholipids, include: PA – phosphatidic acid, PC – phosphatidylcholine, PE – phosphatidylethanolamine, PI – phosphatidylinositol, PS – phosphatidylserine, CL – cardiolipin, and their respective lysospecies: lysoPA, lysoPC, lysoPE, lysoPI and lysoPS. GL – glycerolipids, include: DAG – diacylglycerol, TAG – triacylglycerol. Sterols include: Erg – ergosterol.

Lipid species were annotated according to their molecular composition. Glycero- and glycerophospholipid species are annotated as: <lipid class> <sum of carbon atoms in the fatty acids>:<sum of double bonds in the fatty acids> (e.g., PI 34:1). Sphingolipid species are annotated as: <lipid class> <sum of carbon atoms in the long chain base and fatty acid moiety>:<sum of double bonds in the long chain base and the fatty acid moiety>;<sum of hydroxyl groups in the long chain base and the fatty acid moiety> (e.g. IPC 44:0;4).

### Growth phase experiment

BY4741 was grown in SCglc at 30°C and harvested when OD_600 nm_ reached 1 (sample: OD1), ∼2.7×10^7^ cells/ml, early logarithmic (log.) growth phase; 3.5 (sample: OD3.5), ∼10.5×10^7^ cells/ml, middle log.; 6 (sample: OD6), ∼19.3×10^7^ cells/ml, early stationary and stationary phase (sample: ODstat), 24 h of growth; average OD_600 nm_ 6.6±0.1 SD; ∼20.9×10^7^ cells/ml. Cells were counted using a hemocytometer.

### Media experiment

BY4741 were grown in YPglc, YPraf, YPgly, SCglc and SCraf at 30°C and harvested when OD_600 nm_ reached 1.

### Haploid/diploid experiment

BY4741 (sample: a), BY4742 (sample: α), BY4743 (sample: diploid) were grown in SCglc at 30°C and harvested when OD_600 nm_ reached 1.

### Temperature experiment

BY4741 were grown in SCglc at 15°, 24°, 30° and 37°C (samples: T15, T24, T30 and T37, respectively) and harvested when OD_600 nm_ reached 1.

### Induction experiment

BY4741pGFP (sample: GFP) and BY4741pTM (sample: TM) were grown in SCraf at 30°C and media were exchanged to SCrafgal when OD_600 nm_ reached 0.75. Samples were harvested after 0, 1, 3 and 6 h of induction (OD_600 nm_ reached 0.75, 1, 1.7 and 3.7 for GFP and 0.75, 1, 1.5 and 3.0 for TM, respectively).

### G418 experiment

The *dan2*Δ strain was grown in SCglc (sample: G418-) or SCglc+G418 (sample: G418+) (200 µg/ml) at 30°C and harvested when OD_600 nm_ reached 1.

### Data analysis

Identification of molecular lipid species in mass spectra was performed using LipidXplorer software [19], [20]. Quantification and formatting of the data into a database-compatible format was performed by scripts in the Python programming language developed in house. Lipidomic features were calculated through database queries and adjustably visualized via the Vortex graphical user interface (Dotmatics Ltd.) [21]. Statistical calculations, principal component analysis (PCA) and hierarchical clustering (complete linkage hierarchical clustering on the cosine distance metrics) were done with KNIME data mining software [22].

## Results

### Experimental design

**Figure 1:**
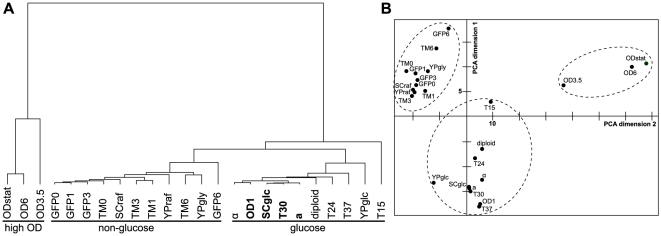
Hierarchical clustering and principal component analysis (PCA) of yeast lipidomes from different growth conditions. A) Hierarchical clustering of yeast lipidomes using lipidomic features (see text) reveals three major subclusters (underlined). Samples grown under “reference conditions” (SCglc in 30°C and OD_600 nm_ = 1) are in bold. B) Principal component analysis of yeast lipidomes using lipidomic features separates three major conditions (encircled with dashed lines) along the first two PCA dimensions. For the abbreviations see Materials and Methods.

Based on experimental protocols commonly applied in yeast research, the influence of various growth conditions on the lipid composition of the wild-type strain BY4741 was investigated by quantitative shotgun mass spectrometry. The tested parameters were: temperature (15, 24, 30, 37°C), carbon source (glucose, raffinose, glycerol), different growth phases (early and middle logarithmic as well as early and stationary phase) complete (YP) vs. synthetic complete (SC) medium, galactose-induced over-expression of a cytosolic as well as a trans-membrane protein and the presence or absence of the selective drug G418. In addition, the lipidome of haploid (a and α) and diploid cells was determined (see Materials & Methods for further details). In order to assess the influence of growth conditions on the lipid composition of yeast cells, it was necessary to ensure that factors such as the handling of the cells, lipid extraction and data acquisition by mass spectrometry were not a source of variations of the yeast lipidome. Therefore, in each experimental run, triplicate control cultures grown under “reference conditions” were included (SC glucose at 30°C and harvested in early logarithmic (log) phase (OD_600_ = 1); corresponding to samples: “OD 1”, “SCglc”, “T30”, and “a” (for details see Materials & Methods)). Variance analyses, as well as hierarchical clustering revealed that the lipidomes of the control samples grown on different occasions (days, media batches) exhibit a remarkable similarity, thus confirming the reliability as well as the consistency of our approach (Fig. 1, Table S1).

### Data analysis

The acquisition of mass spectra of about 80 samples allowed for the absolute quantification of a total of about 160 individual lipid species belonging to 18 lipid classes. The amount of data produced necessitated the development of a suitable approach simplifying and facilitating data analysis and interpretation. To this end, quantitative lipidomic features were derived from the measurements of individual lipid species. Their level of detail ranged from categories through classes down to the species level. For example, quantities of all GP species comprised of two double bonds in their fatty acid moieties were summarized as one lipidomic feature, i.e. GP DB-2. Together with the lipidomic feature GP DB-1 and GP DB-0 (i.e. all GP comprising one or zero double bond, respectively) we obtained a simple expression for the degree of unsaturation of the entire category of GP. We proceeded similarly for the other lipid categories and the lipid classes. Other molecular parameters like FA chain length and hydroxylation were also included in the features. Thus, by way of example, the lipidomic feature PI C-34 summarizes all PI species containing a total of 34 carbon atoms in their hydrocarbon chain.

The absolute quantification of lipid species and the summary of their amounts as lipidomic features provide a quantitative as well as qualitative, functional and biologically relevant description of a lipidome. The calculation of variances of the lipidomic features across the entire sample set or a subset of samples identifies the features that undergo the most pronounced changes. Additionally, the lipidomic features were used for hierarchical clustering of the datasets (see Materials & Methods). This approach simplified the data analysis and interpretation process.

### Influence of different factors on the yeast lipidome - general observations

The quantification of the lipidome of yeast cells grown under the various conditions led to four major observations. First, the carbon source has a strong effect on the overall lipid composition. Lipidomes of cells grown in raffinose and glycerol cluster together, while lipidomes of the glucose-grown cells form a separate cluster (Fig. 1). Second, the progression through the growth phase, i.e. from early logarithmic to stationary phase, causes remarkable changes in the lipidome. This is manifested by the very distant cluster formed by lipidomes of mid-log (OD3.5), early stationary (OD6) and late stationary (ODstat) yeast cultures. Third, there is a pronounced effect of decreasing the growth temperature to 15°C (T15). While the other lipidomes from the growth temperature experiment (T24, T30, T37) group within the “glucose cluster”, the lipidome of cells grown at 15°C appears to be significantly different. Fourth, the galactose-induced overexpression of a cytosolic or a transmembrane protein (within an induction time of up to 3 h) as well as the presence of the selective drug G418 and the different mating types (a, α, diploid) are not causing pronounced changes in the lipid composition of the yeast cells. The lipidomes of galactose-induced cells are part of the “non-glucose cluster”, while the lipidomes of the different mating types cluster tightly with the lipidomes of glucose-grown cells (for a complete dataset, see Table S1). The nature of the changes caused by different carbon sources, growth phase, and growth temperature is presented in the following sections in more detail.

### Temperature

**Figure 2:**
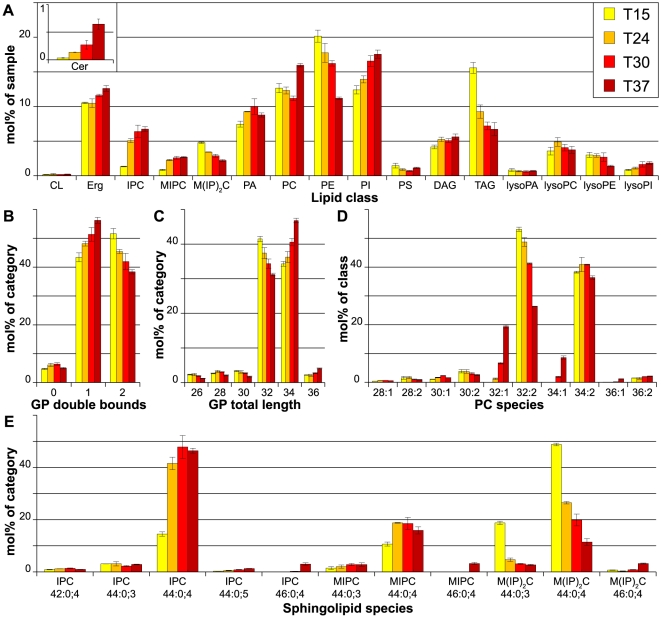
Lipidomics of yeast grown in different temperatures. Yeast cells were grown in SCglc at 15°C (T15), 24°C (T24) 30°C (T30) and 37°C (T37). A) Lipid class composition in mol% of total lipids in the sample. LysoPS and ceramides as minor constituents are not shown. B) Total double bonds of glycerophospholipids (CL omitted for clarity), given as sum of double bounds in fatty acids, in mol% of category. C) Total length of glycerophospholipids, given as sum of fatty acids, in mol% of category. Lysospecies and cardiolipin lengths are omitted for clarity. D) Phosphatidylcholine (PC) species profile in mol% of class. The least abundant species are not shown. E) Sphingolipid species profile in mol% of category. All data were calculated from n = 3 independent samples ± SD. For the lipid nomenclature, sample names and experiment description see Materials and Methods.

The effect of temperature on the lipidome was tested by growing cells at 15, 24, 30 and 37°C. As expected, the degree of unsaturation of the GP showed a gradual trend: it was the highest at 15°C and the lowest at 37°C (Fig. 2 c). This change was mainly caused by a shift from mono- to di-unsaturated GP species. A similar gradual trend was observed for the length of the hydrocarbon chains in GP. While at 15°C GP species with a total sum length of hydrocarbon chains of 32 carbon atoms were most abundant, at 37°C species with 34 carbon atoms became predominant (Fig. 2 d), meaning that the FA chains of the GP species became longer with increasing temperature. On the level of GP classes, the most striking effects were the opposing trends of PI and PE changes. While PE levels decreased with increasing temperature, PI became more abundant (Fig. 2 a). Interestingly, changes in PC abundance did not show a clear temperature-dependent trend. However, changes in its unsaturation index followed the general trend of the other GP, i.e. increase in mono-unsaturated species at the expense of di-unsaturated PCs (Fig. 2 d).

Prominent changes also occurred within the sphingolipids. At 15°C, M(IP)_2_C was the most abundant SP (Fig. 2 a, e), but with increasing temperature its levels decreased, and IPC became dominant. MIPC showed a comparable trend. At the species level, a temperature-dependent increase in the sum length of the hydrocarbon chain moieties (from 44 to 46) was observed (as well as a minor increase in hydroxylation) (Fig. 2 e). Additionally, ergosterol levels also increased gradually, although subtly, with increasing temperature. Furthermore, it is interesting to note that ceramide levels increased significantly at 37°C, while TAG levels increased at 15°C (Fig. 2 a).

### Growth phase

**Figure 3:**
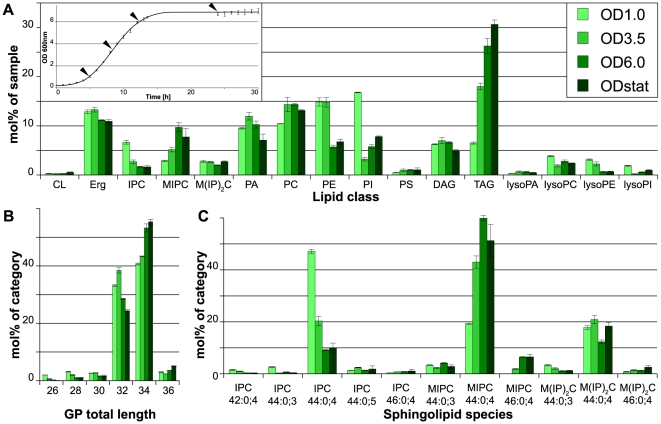
Lipidomics of yeast in different growth phases. Yeast cells were grown in SCglc at 30°C and harvested in early logarithmic (OD1), middle logarithmic (OD3.5) and early stationary (OD6) phase as well as after overnight growth (ODstat). A) Lipid class composition in mol% of total lipids in the sample. LysoPS and ceramides as minor constituents are not shown. Inset: Growth curve with data points indicated by arrowheads. B) Total length of glycerophospholipids in mol% of category. Lysospecies and cardiolipin lengths are omitted for clarity. C) Sphingolipid species profile in mol% of category. All data were calculated from n = 3 independent samples ± SD. For the lipid nomenclature, sample names and experiment description see Materials and Methods.

The lipidomes of yeast cells from different growth phases were quantified. Samples were withdrawn in early (OD1), middle logarithmic (OD3.5) and early stationary phase (OD6) as well as from stationary phase after 24 h of continuous growth (ODstat; Fig. 3 a, inset). During progression through the growth phases, a strong increase in TAG levels was observed. Interestingly, the accumulation of TAG was already more than 2-fold in the mid-log phase when compared with the early log phase and increased further in the later growth phases (Fig. 3 a). Concurrently with the increase of TAG, the PI level dropped by more than 75% from early to mid-log phase, while the PE level dropped by 50% after mid-log phase. PI levels recovered slightly in later growth phases, while PE levels stabilized. The PC level remained unchanged throughout the course of growth. However, the unsaturation of PC was strongly decreased already in mid-log phase and there was a pronounced increase in its hydrocarbon chain length in stationary phase (Table S1). On average, the length of GP increased with progression through the growth phases (shift from C32 to C34 and also a small but significant increase of C36), while the unsaturation index remained unchanged (Fig. 3 b).

With respect to the complex sphingolipids, IPC is the most abundant SP in early log phase. However, the IPC level greatly decreased in mid-log and further in later growth phases. Concurrently with the drop in IPC, MIPC becomes the most abundant sphingolipid, while the M(IP)_2_C level remained constant throughout the experiment (Fig. 3 a). Moreover, while progressing through the growth phases, an increase in the length of the hydrocarbon chain moieties (from 44 to 46) was observed. Additionally, the hydroxylation of the SP subtly increased (Fig. 3 c). These changes were similar to those observed in the temperature experiment.

### Carbon source

We investigated the influence of the carbon source in the growth medium on the yeast lipidome by growing the cells in complete medium (YP, see Materials & Methods) supplemented with glucose, raffinose (YPglc and YPraf, resp.; fermentable carbon sources) or glycerol (YPgly, non-fermentable carbon source).

**Figure 4:**
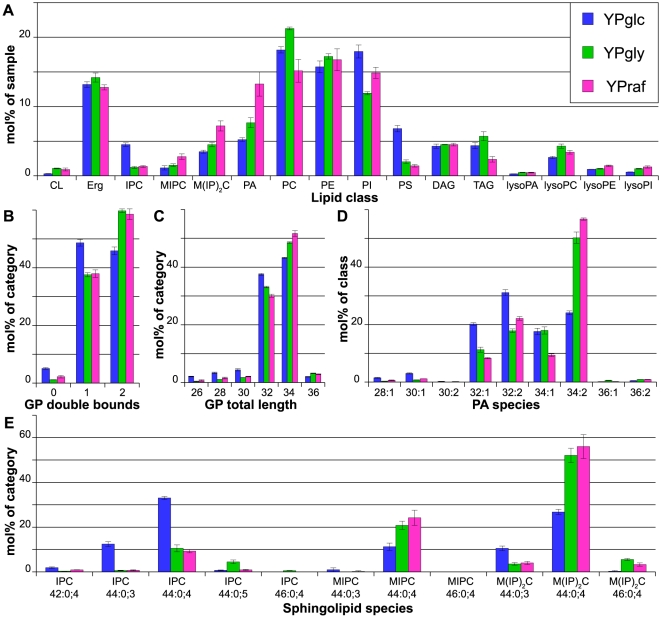
Lipidomics of yeast grown on different carbon sources. Yeast cells were grown at 30°C in full medium (YP) supplemented with different carbon sources (glucose: YPglc; raffinose: YPraf; glycerol: YPgly) A) Lipid class composition in mol% of total lipids in the sample. LysoPS and ceramides as minor constituents are not shown. B) Total double bonds of glycerophospholipids (CL omitted for clarity), given as sum of double bounds in fatty acids, in mol% of category. C) Total length of glycerophospholipids, given as sum of fatty acids, in mol% of category. Lysospecies and cardiolipin lengths are omitted for clarity. D) Phosphatidic acid (PA) species profile in mol% of class. E) Sphingolipid species profile in mol% of category. All data were calculated from n = 3 independent samples ± SD. For the lipid nomenclature, sample names and experiment description see Materials and Methods.

Hierarchical clustering revealed that the lipidomes of glycerol- and raffinose-grown cells are similar to each other, but differ from the lipidome of glucose-grown cells (Fig. 1 a). Interestingly, a significant increase in cardiolipin could be observed in cells grown in YPraf and YPgly (Fig. 4 a). Furthermore, PS was strongly elevated in YPglc, while PA was increased in YPraf and also in YPgly. The abundances of PC and PI also changed depending on the carbon source, while the PE levels were not affected. In general, GP became more unsaturated (to a large extent due to an increase in di-unsaturated PS and PA species, Fig. 4 b, d) and had longer hydrocarbon chains (increase of C34 species at the expense of C32, Fig. 4 c) in raffinose- and glycerol-containing media.

The carbon source also had an impact on the sphingolipids. In glucose medium, IPC was the most abundant SP class. However, in YPraf as well as in YPgly M(IP)_2_C was predominant (Fig. 4 e). Moreover, the hydroxylation state of the SP was elevated in glycerol-grown cells.

Additionally, it was interesting to note that differences in the lipid composition could be observed between cells grown in complete (YP) and synthetic complete (SC) medium, irrespective of the carbon source (Table S1).

### Flexibility of the yeast lipidome

During the course of the experiments we observed that some lipidomic features (e.g. lipid classes or unsaturation indices) varied to a larger extent and more often than others, depending on growth conditions. This observation prompted us to derive general rules about the potential variability of the lipids. To describe the variability of lipidomic features, here we introduce the term flexibility. We define flexibility as the index of dispersion (variance-to-mean ratio, VMR) of a given lipidomic feature across the entire dataset, i.e. changes of its abundance in different conditions. A VMR<1 indicates that data are under-dispersed or, in other words, the abundance of a lipidomic feature deviates from the mean only to a small extent. Thus, a VMR<1 indicates low flexibility of a given feature. On the other hand, a VMR>1 indicates that the data are over-dispersed and have a clumped distribution, that is, the abundance of a lipidomic feature can deviate strongly from the mean. Therefore, the VMR is a useful measure of the capability of a lipidomic feature to undergo quantitative changes (i.e. of its flexibility).

The analysis of the flexibility of the lipid categories and classes revealed that GL is the most flexible lipid category, exhibiting the highest VMR (Table 1). This is mainly due to the high flexibility of TAG. Notably, the least flexible lipid categories are the SP and the sterols (Erg), while the GP flexibility is intermediate.

**Table 1:** Flexibility of the lipid categories and classes.

Lipid	Category	VMR
TAG	GL	5.96
**GL**		**3.98**
PS	GP	1.44
IPC	SP	1.37
PI	GP	1.20
MIPC	SP	1.09
PE	GP	0.93
**GP**		**0.89**
MIP2C	SP	0.63
PC	GP	0.61
PA	GP	0.48
**SP**		**0.22**
CL	GP	0.20
Erg	ST	0.16
Cer	SP	0.12

The flexibility is expressed as the variance-to-mean ratio (VMR). For details see text.

Interestingly, when comparing the flexibility of the complex SP classes IPC, MIPC and M(IP)_2_C with the flexibility of the SP category as such, it turned out that the classes constituting the category are more flexible than the category itself. Thus, the total average amount of complex SP was stable, while the relative abundance of the SP classes within the category was highly variable.

The most flexible GP class is PS (Table 1). This is largely due to the strong increase of the PS level in cells grown in YPglc (Fig. 4 a). The next most flexible classes are PI, PE, PC, and PA. CL is the least flexible GP class, since its abundance changed to a small extent only when glycerol or raffinose was used as carbon source.

### Internal variability of lipid classes

Each lipid class may also be characterized by an internal variability. The internal variability describes the extent of change within a lipidomic feature (e.g. the average variation of the length, unsaturation index, or hydroxylation within a class). Therefore, the internal variability is a measure for the extent of changes that occur in the species profile of a given lipid class.

**Figure 5:**
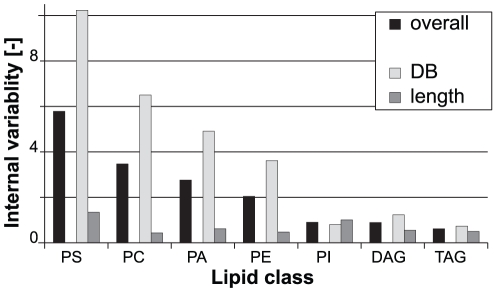
Internal variability of lipid classes. The internal variability was calculated by averaging the variance-to-mean ratio (VMR) of the length and DB features of the respective lipid classes across the entire sample set (“overall”). Individual calculation of the average VMR of the length and DB features, respectively, reveals that the overall internal variability of a lipid class is largely determined by changes in the DB features (“DB” vs. “length”).

For the description of the internal variability of the individual GP classes, the VMR of the length and unsaturation features of each class were averaged in order to obtain an estimation of the extent of the qualitative changes within the class. For example, it was observed that PC exhibits much stronger changes in its species profile than PI (Table S1). Aiming at the expression of this observation by a single numerical value (the internal variability index), the VMR of the hydrocarbon chain length and the unsaturation indices within the respective classes (i.e. PI and PC) were calculated and averaged for each class. It turned out, that PC shows a higher internal variability than PI (Fig. 5), confirming the observation in the species profiles. Therefore, the internal variability appears to be an appropriate measure for the qualitative changes that occur within a given lipid class or category.

Further analysis of the internal variability indices revealed that PS is the most variable GP class (Fig. 5). Again, this is mainly due to the strong changes in PS in yeast grown in YPglc. Apart from that, PA and PC appear to have large internal variability, while PE and PI are rather invariable, with PI being the least variable GP class (except for CL, which was omitted from this analysis, since in the majority of experiments only a few species could be detected). Interestingly, except for PI, the internal variability of all other GP classes is largely determined by the unsaturation index (Fig. 5). Furthermore, the GP classes are internally more variable than the GL classes TAG and DAG.

## Discussion

Here, we present for the first time a detailed (≈95% lipidome coverage; [1]), quantitative, mass spectrometry-based assessment of the flexibility of a eukaryotic cell's membrane lipidome under a variety of conditions. Individual species abundances were clustered into defined lipidomic features. By calculating the VMR of the lipidomic features, we obtained a simple expression that describes the extent of alterations that are induced by changing conditions. This approach simplified the interpretation of the large lipidomic dataset and revealed important insights into the inherent flexibility of the yeast lipidome.

### Implications for experimental practice

Even minor changes of the growth conditions can have a profound influence on the lipidome of yeast cells. Not surprisingly, the most pronounced changes are induced by changing temperature and growth phase, but also by different carbon sources. These changes might thus affect lipid-dependent phenotypes under investigation. For example, the multiple changes in the lipidome that occur already between the early- and mid-logarithmic growth phase in the course of 3–4 hours (Fig. 3) might easily lead to misinterpretation of data when the growth phase of different strains is not tightly controlled or when mutant and wild-type strain differ greatly in terms of growth dynamics. This fact might deserve consideration especially in high throughput approaches using yeast cells or cell culture lines where synchronization of growth phases can be particularly challenging. We also note that PS amounts and its species profile can vary strongly due to prolonged sample washing and handling after cultivation (our unpublished observations). Therefore, changes observed regarding PS should be interpreted with caution with respect to their biological significance. Of course, other factors not investigated here might have to be taken into account as well.

### Temperature and growth phase effects on the cellular lipidome

It is known that decreasing the growth temperature leads to an increase in FA unsaturation as well as shortening of the FAs [1], [23], which the present study confirmed. Higher growth temperatures evoke the expected changes in hydrocarbon chain properties of the GP. Along with the increase in longer and more saturated fatty acids, the ergosterol content is elevated at higher growth temperatures [24], [25]. These changes probably reflect adjustments of membrane fluidity to the different growth temperatures. Similarly, the opposing trends of PI and PE abundances might serve to maintain membrane properties in a physiologically optimal state. The small headgroup and the unsaturated hydrocarbon chains give rise to the conical shape of the PE molecule, which results in inverted hexagonal (H_II_) phase present in pure PE membranes [26]. Therefore, PE-rich bilayers are in a state of high curvature frustration, resulting in elevated membrane fluidity [27]. On the contrary, the PI molecule with its inositolphosphate headgroup can form intermolecular hydrogen bonds, which have a condensing effect on the bilayer [28]. The low degree of PI unsaturation further supports this effect [29]. Thus, an interchange between the structurally opposing PE and PI molecules might provide an efficient means to fine-tune membrane properties at varying temperatures.

The most pronounced effect caused by the progression through the growth phase was the accumulation of TAG. It is a well-established fact that when approaching stationary phase, yeast cells start to accumulate TAG, which help them to sustain periods of starvation and serve as a reservoir for FAs that are needed for the synthesis of membrane lipids when growth is resumed [10], [30]. The accumulation of TAG occurs at the expense of GP, which is in agreement with the observation that the expression of GP synthesizing genes is repressed when cells enter the stationary phase [31]. While the abundance of PC is hardly affected by the growth phase, PI and PE reduction is the strongest, resulting in PC becoming the most abundant GP in the early stationary phase. Differences in growth phases might thus explain the different results concerning the question which GP is the most abundant in yeast cells [1], [32]. De Kroon and colleagues speculated that PC is preferentially used in stationary cells because it is a “low maintenance GP”, in the sense that it forms ideal bilayers and has a low turnover rate, while PE as a non-bilayer forming lipid is needed in periods of physiological activity [33]. This reasoning could be extended to PI, for the reasons discussed above. Furthermore, we observed a strong decrease in the unsaturation of PC, while the effect for the remaining major GP was modest (PI) or not observable at all (PE). Therefore it appears that since the membrane properties in later growth phases are largely determined by PC, fine-tuning is achieved by remodeling the acyl chain composition within this particular lipid class [34].

### The metabolic state of a cell is reflected by its membrane lipidome

The overall yeast lipid composition is under control of the glucose repression mechanisms [35], as evident from the fact that hierarchical clustering revealed that lipidomes from cells grown in glucose as carbon source form one cluster, while lipidomes of non-glucose-grown cells form another one (Fig. 1). Therefore, the presence of glucose as a carbon source is the major differentiating factor for a lipidome. Under aerobic conditions in early growth phases, glucose is catabolized mainly by glycolysis and fermentation. Aerobic fermentation is less efficient for raffinose, probably due to lower uptake rates by sugar transporters, thus reducing the flux through glycolysis [36]. Glycerol, however, is considered a non-fermentable carbon source, since yeast cells cultured in glycerol-containing media require functional mitochondria for growth. Thus, ATP production in the presence of glycerol as the sole carbon source is accomplished by respiration/oxidative phosphorylation. It has long been known that the GP content of yeast cells correlates with their respiratory activity [37], [38]. The major changes observed were an increase in CL (reflecting the proliferation of mitochondria required for respiration [39], [40]), PE and GP unsaturation [33], [41], [42], while PI/PS (which could not be distinguished with the method used then) decreased [39]. Here, these observations are confirmed and extended. In particular, the overall increase in GP unsaturation is largely due to the increased unsaturation of PA, PS and PE. The increase in unsaturation of PA and PS might lead to a sufficiently unsaturated pool of precursors for PE synthesis in the mitochondria by decarboxylation of PS [42], [43]. The idea that PS serves as a source for unsaturated PE is supported by the overall reduction of PS in yeast cells grown in non-glucose carbon sources. However, other effects leading to a reduction of PS amounts cannot be excluded.

How could the lipid composition be regulated by the available carbon source? The regulation of the metabolism in response to glucose availability is mediated by the Ser/Thr-kinase Snf1p [35]. Interestingly, mutant alleles of the gene encoding the Snf1p phosphatase Reg1p interact genetically with the ino4-8 allele [44]. Ino4p is a transcriptional activator that, together with Ino2p, controls the expression of genes encoding phospholipid-synthesizing enzymes [45]. Therefore, a connection between the glucose response pathway and the Ino2/4 regulon may explain the differences in the lipidomes of cells grown in glucose or non-glucose carbon sources. In addition, the expression of the PI synthase gene PIS1 is also regulated in a carbon source-dependent manner [46].

Taken together, the data provided here illustrate that the membrane lipid composition reflects the metabolic state of a cell (i.e. fermentation vs. respiration). In analogy to what has been observed with respect to changes of cellular morphology in epithelial cells [2], this does not apply to a particular lipid class or species only, but to the entire lipidome, since a multitude of differences can be observed. Therefore, lipidomics may prove a useful multi-parametric (and therefore robust), quantitative read-out to monitor the metabolic state (but also the morphological state) not only of a cell, but also of tissues and multicellular organisms.

### Flexibility of the yeast lipidome

In order to evaluate the biological significance of statistically significant changes in the cellular lipid composition evoked by either changes in the growth conditions or by genetic manipulation, it is essential to understand the intrinsic natural range of flexibility of the lipidome. To this end, the VMR of lipidomic features induced by different growth conditions was used as a quantitative and unbiased expression for the flexibility and the internal variability of the lipid classes.

The most flexible lipidomic feature across the growth conditions tested in the present study, were the glycerolipids (Table 1). Their flexibility was to a major extent determined by TAG. None of the membrane lipid classes exhibited a comparable flexibility. This finding is in line with the view, that TAG serve as a reservoir for membrane constituents (i.e. fatty acids) in times when membrane lipid synthesis is decreased [10], [30]. The high flexibility of TAG argues for two things: First, the flexibility of the membrane lipid composition is limited by the need to maintain membrane homeostasis. Second, TAG abundance can vary strongly, since they are not membrane-forming lipids and thus do not contribute to the overall membrane properties.

In contrast to the flexibility of TAG, the abundance of ergosterol and SP is rather stable (Table 1). Interestingly, while the abundance of the SP category as a whole is comparatively conserved, the abundance of the inositol phosphate-containing SP IPC, MIPC and M(IP)_2_C, is subject to great variation. This suggests that the SP classes share a common feature that is essential for the cell. This may be the ceramide backbone, the very long chain fatty acids (VLCFA) and/or the inositol phosphate-containing headgroup. Accordingly, synthesis of these structural components is an essential requirement for the viability and growth of yeast cells [47]–[50]. However, yeast cells can survive without SP synthesis in the presence of a suppressor mutation (*SLC1-1*) that enables synthesis of VLCFA-containing PI, which can be further mannosylated [51], [52]. In this strain, SP-dependent processes are functional [53], . Hence, common essential traits of the yeast SP could be the VLCFA together with the inositol phosphate-containing headgroup.

Further analysis of the flexibility of lipidomic features revealed that the unsaturation index of the GP possesses much stronger variation than the fatty acid chain length (Fig. 5). This is the case for PC, PE, PS and PA (but surprisingly not for PI). The effect of changing the degree of unsaturation of a GP on its physical properties (e.g. phase transition temperature) is much stronger than changing the length of the hydrocarbon chains [55]. Thus, adjusting the membrane fluidity by means of unsaturation is more efficient than changing the fatty acid length and therefore this is the predominant mechanism in living cells [56]. The evidence provided here implies that this holds true also for yeast cells. Additionally, while regulating the unsaturation of the GP is the primary mechanism for the adjustment of membrane properties, varying the fatty acid chain length might be a secondary means to achieve further, subtler fine-tuning. Along these lines, the flexibility of the FA chain length is limited by the need to maintain a critical membrane thickness for the accommodation of transmembrane proteins. Moreover, (phospho)lipids with short FA are relatively water soluble, resulting in high on-off rates and the fluid phase of membranes consisting of short chain lipids is thermodynamically unstable [55]. Thus, adjusting the unsaturation is an efficient instrument for tuning bilayer properties without compromising the essential functions of the membrane.

Among the GP classes, PA exhibits a remarkably high degree of internal variability (i.e. the variation of features within a given lipid category or class). This is interesting to note, since PA as the entry point into the GP biosynthetic pathways, can be seen as a moderator between the free FA pool and the FA bound to GP and in this way is a checkpoint for the final FA composition of GP [56]. PC behaves in a comparable way; its abundance is conserved in different conditions, while its species profile varies. This might be caused by different substrate specificities of the two alternative PC biosynthetic pathways (Kennedy pathway vs. methylation of PE) as well as by acyl chain remodeling [34], [57], [58]. On the contrary, the PI species profile is very conserved. In fact, it is the only GP class in which the unsaturation index is less flexible than the hydrocarbon chain length (Fig. 5). The reasons for this might be a tight substrate specificity of the PI synthase Pis1p and a low degree of acyl chain remodeling. Additionally, this implies that monounsaturation (PI 32:1 and PI 34:1) is critical for the function of PI species. However at the same time, the PI abundance is very flexible. Therefore, it appears that the modulation of membrane properties relies on the headgroup, rather than on acyl chain remodeling of PI.

Since the present study focuses on a set of commonly applied yeast growth conditions, it is obvious that the generality of the flexibilities of the different lipidomic features described here can only be proven by further experiments. Moreover, it should be noted that the observed flexibility and internal variability of the lipid classes might also be caused by changes in the abundance and/or structure of subcellular organelles. It will be interesting to see how other lipid classes like phosphoinositides are affected and how the cell achieves its lipidomic flexibility. Changes in transcript levels as well as abundance and activity of lipid metabolic enzymes are likely to be involved, leading to altered fluxes through the different branches of the lipid biosynthetic pathways.

### Conclusions, perspective

Lipidomics is emerging as a widely used approach to study the relevance of lipids in a variety of pathological conditions such as obesity, diabetes, cardiovascular disease and cancer [59]. Interpretation as well as evaluation of changes in a lipidome caused by (or being causative for) certain disease states requires the knowledge of the intrinsic natural variability of a given lipidome. Therefore, by using a lipidomic dataset obtained from yeast cells grown under a variety of different conditions, we set out to identify a simple, yet quantitative and unbiased expression of the flexibility of a eukaryotic lipidome and its features. This approach has proven useful since it enabled the confirmation of the high flexibility of features such as cellular triacylglycerol content and the unsaturation index of the membrane constituting glycerophospholipids. Furthermore, it demonstrated novel characteristics of the yeast lipidome, e.g. the low flexibility of the sphingolipid and ergosterol abundance.

Finally, the flexibility of a lipidome (or any other “ome” that can be assessed quantitatively) may be envisioned as a novel parameter for the screening of the physiological state of a cell or an organism, especially under conditions where biotic as well abiotic factors cannot be tightly controlled.

## Supporting Information

Table S1 **Complete dataset of the lipid composition of yeast cells grown under the various conditions (given as mean in mol% of total lipids; ± standard deviation, n = 3).**
(CSV)
